# Ozone-assisted catalytic oxidation of aqueous nitrite ions on HZSM-5 zeolites

**DOI:** 10.1038/s41598-019-50662-7

**Published:** 2019-10-04

**Authors:** Mengyue Ying, Mengdi Zhang, Yue Liu, Zhongbiao Wu

**Affiliations:** 1 Department of Environmental Engineering, Zhejiang University, Hangzhou, 310027 P.R. China; 2 Zhejiang Provincial Engineering Research Center of Industrial Boiler & Furnace Flue Gas Pollution Control, Hangzhou, 310027 P.R. China

**Keywords:** Pollution remediation, Materials science

## Abstract

Simultaneous removal of NO_*x*_ and SO_2_ during the wet absorption process has made it possible for nitrogen resource utilization. However, nitrites formation at high ratio in absorption solution would limit its application. In this study, the catalytic oxidation behaviors of aqueous nitrite ions assisted by ozone on HZSM-5 zeolites with different SiO_2_/Al_2_O_3_ ratios have been investigated. The experimental results revealed that the oxidation and disproportionation reactions of nitrite ions took place competitively, both of which were accelerated under acidic condition. Moreover, the introduction of HZSM-5 zeolites and ozone would significantly improve the nitrite oxidation rate, where the zeolites with high SiO_2_/Al_2_O_3_ ratios were found to be more effective owing to the enhanced adsorption of nitrite ions and ozone. Based on the results under different operating conditions (such as O_3_ concentration, HZSM-5 dosage, pH values and presence of radical scavengers etc.), the reaction mechanism was then proposed. The disproportionation reaction of nitrite ions mainly occurred in the bulk solution. And the catalytic oxidation of nitrite ions over zeolites proceeded via a non-radical surface reaction between the adsorbed nitrite ions and ozone/oxygen molecular.

## Introduction

Nitrogen oxides (NO_*x*_) caused by fossil fuel combustion are one of the major atmospheric pollutants and can result in acid rain and photochemical smog^1–3^. The selective catalytic reduction with ammonia (NH_3_-SCR) is the most commercialized control technology for stationary-source NO_*x*_ emission^4,5^. However, in order to meet China’s ultra-low emission standards related to NO_*x*_ (50 mg/Nm^3^) for power plants^6^, excessive ammonia injection during NH_3_-SCR process has been executed commonly, which may bring out some problems like severe ammonia leakage and blockage of air pre-heater. Furthermore, the NH_3_-SCR technology seems to be not very cost-effective for low-content NO_*x*_ abatement from industrial boilers and furnaces. As such, Wet scrubbing of NO_*x*_ in flue gas desulfurization (FGD) device, due to its low costs and less land occupied, has been considered as a promising supplementary or alternative to the NH_3_-SCR technology^7^.

When combined with additional oxidation processes (transforming NO to NO_2_, normally), NO_*x*_, as well as SO_2_, could be effectively removed in wet FGD device via a series of free radical reactions between NO_2_ and sulfite ions^8^. As the main absorption products of NO_*x*_ scrubbing are nitrites and nitrates^9–11^, one of the major disadvantages of wet NO_*x*_ absorption processes is the disposal of waste water containing large amounts of nitrites and nitrates, which requires high additional cost. In addition, aqueous nitrites have carcinogenic effects and may release NO to gas phase causing secondary pollution^12–14^. Especially, the nitrites are extremely unstable under acidic conditions due to the speedy disproportionation reaction of NO_2_^−^ (Eq. (1))^15–17^. By contrast, nitrates, which are more stable, could be used as industrial or fertilizer raw materials^18^ and phase change materials^19^, etc. Hence, exploring appropriate ways to oxidize NO_2_^−^ to NO_3_^−^ in the waste water of wet denitration processes is vital for both possible nitrogen resource recovery and secondary pollution controlling.

1 $$3N{O}_{2}^{-}+2{H}^{+}=2NO+N{O}_{3}^{-}+{H}_{2}O$$

NO_2_^−^ can be oxidized by strong oxidizers like ozone^20^, hydrogen peroxide^21^, sodium hypochlorite^22^, etc. As the cost of strong oxidizing agents is relatively high, these processes may bring additional economic pressure. Biological technology also could be employed for converting NO_2_^−^ to NO_3_^−^ by nitrification process^23–25^. However, it would be normally a time-consuming process^26^. To date, heterogeneous catalytic wet oxidation has been considered as an effective technology to remove both organic and inorganic pollutants from industrial waste water. Generally, such technology is of the advantages including low cost, mild operational condition, low energy consumption and high efficiency^27–29^. Heterogeneous catalytic wet oxidation processes usually use oxygen or air as oxidants, sometimes strong oxidizers like ozone are also added to assist the oxidation process^30–32^.

As reported, various solid catalysts for heterogeneous wet catalytic oxidation have been investigated, such as zeolites^33^, metal exchanged zeolites^26^, carbon materials^34^, metal oxides^26,27,35^ and precious metals based catalysts^36^. The reaction mechanisms include adsorption, direct oxidation, and free radical oxidation^37^. Among the catalysts investigated, ZSM-5 zeolites, due to its large specific surface area, special pore-structure and outstanding thermal/hydrothermal stability^38,39^, have been widely used for aqueous catalytic oxidation. According to the study of Ikhlaq and coworkers, ZSM-5 zeolites could effectively enhance the oxidant adsorption, thus facilitating the catalytic oxidation reaction^40^. Furthermore, zeolites were also used as adsorbents for nitrites removal^41^. As such, ZSM-5 zeolites could be potential superior catalysts for catalytic wet oxidation of nitrite ions.

In this study, HZSM-5 zeolites with different SiO_2_/Al_2_O_3_ ratios were employed for the catalytic oxidation of NO_2_^−^ by air. And the addition of trace ozone was used to improve the oxidation of NO_2_^−^ under ambient condition. The goal of this study was to evaluate the activities of NO_2_^−^ oxidation on various HZSM-5 zeolites under different operating parameters and elucidate the reaction behaviors including NO_2_^−^ oxidation and disproportionation. Furthermore, the reaction mechanism was discussed in detail.

## Experimental Methods

### Materials and reagents

Tianjin Nanhua Catalysis Co., Ltd. (Tianjin, P. R. China) provided HZSM-5 zeolites with different SiO_2_/Al_2_O_3_ ratios in power form. Sodium nitrite were obtained from Macklin Inc (Shanghai, P. R. China). All chemicals were of analytical grade and were used without further purification.

### Catalyst characterization

After being pretreated at 100 °C under vacuum, the specific surface area, pore volume and pore size of ZSM-5 zeolites used in this study were investigated by the BET-BEJ method on a nitrogen adsorption apparatus (JW-BK132F, China).

The points of zero charge for ZSM-5 with different SiO_2_/Al_2_O_3_ ratios were determined by salt addition method as described by Mustafa^42^. The pH of 0.1 M NaNO_3_ solution was adjusted to 3, 4, 5, 6, 7, 8, 9 and 10 at 293 K using sulfuric acid and sodium hydroxide. Then 1.0 g ZSM-5 (SiO_2_/Al_2_O_3_ ratio = 18, 60, 130, 200 and 360, respectively) was added into each beaker containing 100 ml solution and the mixture was stirred to mix well. After resting for 36 h, the final pH of each beaker was recorded. ∆pH (the difference between initial pH and final pH) was plotted against initial pH values and the PZCs values were the pH values where ∆pH is zero.

### Experimental system

#### Oxidation experiments

The oxidation experiments were carried out in a batch reactor (diameter 80 mm; height 120 mm) under atmospheric pressure. The aqueous solution (200 ml with concentration of NO_2_^−^ was 100 mg/L) containing zeolites was added to the reactor which was put in the water bath in order to maintain the temperature of 30 °C and the mixture was stirred during the whole experiment. The pH value of solution was adjusted by using 0.1 M solution of sulfuric acid and sodium hydroxide. Ozone was generated from pure oxygen by AZCO HTU-500E ozone generator (America) and was continuously mixed with the gas flow to maintain the target concentration. The different oxygen partial pressure was maintained by adjusting the ratio of pure oxygen to nitrogen. The mixture flow (1.5 L/min) was bubbled though a glass cube (inner diameter is 4 mm) at the bottom of the reactor. The period of each oxidation experiment was 90 min. Samples were drawn after every 15 min and then were filtered (PTEE 0.22 μm springe filter) before testing. Moreover, 10 mg/L of p-benzoquinone (BQ) or tertiary butyl alcohol (TBA) were added into the solution when used.

#### Adsorption experiments

The adsorption experiments were conducted to investigate the adsorption capacity of ZSM-5 zeolites towards NO_2_^−^. 1 g zeolites (SiO_2_/Al_2_O_3_ = 360) were added into 200 ml solution (100 mg/L NO_2_^−^) with pH value of 3, 5, 7, 9 and 11, respectively. The mixture was continuously stirred for 1 min and then was rested for another 9 min at 30 °C. Samples were collected at 1, 3, 6 and 10 min followed by filtration (PTEE 0.22 μL springe filter).

### Analytical method

The concentration of ozone in the mixture flow was determined by the IN2000-L2-LC ozone analyzer (INUSA, America).

The concentrations of NO_2_^−^ and NO_3_^−^ were determined by ion chromatography using the 850 Professional IC Anion MCS system. A Metrosep A Supp 5 column (250 mm L × 4.0 mm ID) with integrated chip and an IC conductivity detector (Metrohm, Switzerland) were used in the determination. The injection volume of the sample was 20 μL and analyses were performed at a flow rate of 0.7 ml/min. The total NO_2_^−^ conversion at 90 min (NC-T) was calculated as

2 $$NC-T( \% )=\frac{{C}_{0-N{O}_{2}^{-}}-{C}_{90-N{O}_{2}^{-}}}{{C}_{0-N{O}_{2}^{-}}}\ast 100 \% $$

the NO_2_^−^ conversion via oxidation reaction (NC-OR) was calculated as:

3 $$NC-OR( \% )=\frac{3\ast {M}_{N{O}_{2}^{-}}\ast ({C}_{90-N{O}_{3}^{-}}-{C}_{0-N{O}_{3}^{-}})-{M}_{N{O}_{3}^{-}}\ast ({C}_{0-N{O}_{2}^{-}}-{C}_{90-N{O}_{2}^{-}})}{2\ast {C}_{0-N{O}_{2}^{-}}\ast {M}_{N{O}_{3}^{-}}}\ast 100 \% $$

the NO_2_^−^ conversion via disproportionation reaction (NC-DR) was calculated as:

4 $$NC-DR( \% )=\frac{3}{2}\ast \frac{{M}_{N{O}_{3}^{-}}\ast ({C}_{0-N{O}_{2}^{-}}-{C}_{90-N{O}_{2}^{-}})-{M}_{N{O}_{2}^{-}}\ast ({C}_{90-N{O}_{3}^{-}}-{C}_{0-N{O}_{3}^{-}})}{{C}_{0-N{O}_{2}^{-}}\ast {M}_{N{O}_{3}^{-}}}\ast 100 \% $$

The proportion of disproportionation reaction (η) was calculated as:

5 $${\rm{\eta }}( \% )=\frac{3}{2}\ast [1-\frac{{M}_{N{O}_{2}^{-}}\ast ({C}_{90-N{O}_{3}^{-}}-{C}_{0-N{O}_{3}^{-}})}{{M}_{N{O}_{3}^{-}}\ast ({C}_{0-N{O}_{2}^{-}}-{C}_{90-N{O}_{2}^{-}})}]\ast 100 \% $$

where ${C}_{0-N{O}_{2}^{-}}$ and ${C}_{90-N{O}_{2}^{-}}$ (mg/L) are the initial and the last (at 90 min) concentration of NO_2_^−^, respectively; ${C}_{0-N{O}_{3}^{-}}$ and ${C}_{90-N{O}_{3}^{-}}$ (mg/L) are the initial and the last (at 90 min) concentration of NO_3_^−^, respectively; ${M}_{N{O}_{2}^{-}}$ and ${M}_{N{O}_{3}^{-}}$ (mg/mmol) are the molar mass of NO_2_^−^ and NO_3_^−^, respectively.

## Results and Discussion

### NO_2_^−^ conversions during different oxidation processes

**Figure 1:**
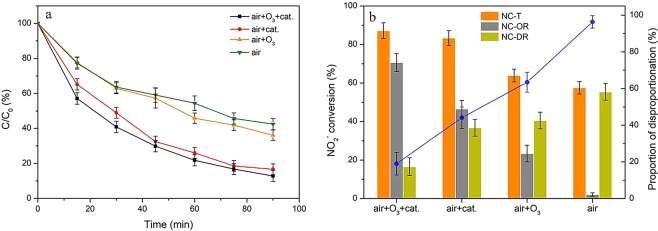
The variations in NO_2_^−^ concentration (**a**) and the related conversions via oxidation and disproportionation reactions (**b**) under different oxidation processes. Reaction conditions: C_0_ = 100 mg/L, gas flow rate = 1.5 L/min, [O_3_] = 100 ppm (if use), T = 30 °C, pH = 3, catalyst dosage = 0.2 g (if use), V = 200 ml, SiO_2_/Al_2_O_3_ = 360.

Figure 1 showed the variations of nitrite ions content with 90 min at pH 3.0 during different oxidation processes and the related conversions via different reaction pathways (oxidation or disproportionation reactions), respectively. It could be clearly seen that the NO_2_^−^ conversions were greatly enhanced after HZSM-5 zeolites addition. For instance, the total NO_2_^−^ conversion was around 57% by using air as the oxidant without HZSM-5 catalyst, while that was over 80% with HZSM-5 catalyst addition. Furthermore, it was found that the selectivity to oxidation reaction was also significantly improved after using HZSM-5 catalyst. Without catalyst, air could hardly oxidize NO_2_^−^ to NO_3_^−^, and the disproportionation reaction (more than 96%) dominated the total NO_2_^−^ converting process. However, with the introduction of HZSM-5 catalyst, the proportion of disproportionation reaction was decreased to ca. 44%. This fact indicated that the oxidation reaction of NO_2_^−^ by oxygen mainly proceeded on the surface of catalysts, which may be resulted from the adsorption of nitrite ions^41^ and oxygen^43^ on zeolites. Additionally, it was found that the addition of trace ozone considerably improved the oxidation of NO_2_^−^ both in the presence and absence of the catalyst. As a stronger oxidant than oxygen, ozone could directly react with nitrites to form nitrates^44,45^. And HZSM-5 zeolites could further provide sufficient reaction interfaces for ozone and nitrite ions, thus resulting in significantly enhanced oxidation efficiency. As such, the highest conversion by oxidation (71%) was obtained in the HZSM-5/air + O_3_ process.

### Effects of pH values

**Figure 2:**
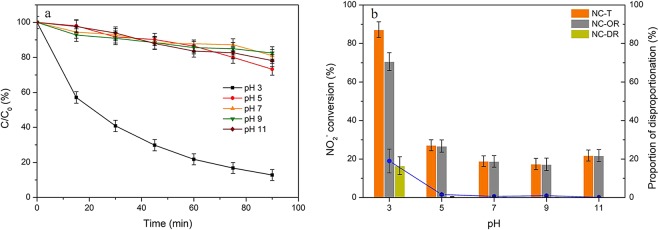
Effects of initial pH value on (**a**) NO_2_^−^ content variations and (**b**) the related conversions via oxidation and disproportionation reactions. Reaction conditions: C_0_ = 100 mg/L, gas flow rate = 1.5 L/min, [O_3_] = 100 ppm, T = 30 °C, catalyst dosage = 0.2 g, V = 200 ml, SiO_2_/Al_2_O_3_ = 360.

Figure 2 showed the effects of initial pH values on the conversions of NO_2_^−^ on HZSM-5 zeolites. It was found that catalytic oxidation reactions were pH sensitive. The total conversion efficiency of NO_2_^−^ at pH 3 was extremely high (more than 88%), while they were all around 20% at other pH values. And the disproportionation of NO_2_^−^ was almost not detected at pH value higher than 5.0, which may be due to the fact that there existed little nitrous acid in the solution since the p*K*a value of nitrous acid is 3.29^46^. As is known, the pH_PZC_ of the catalyst and the pH value of solution can determine the surface charge properties of ZSM-5 zeolites, as the surface always is covered by hydroxyl groups^47^. When the pH of solution is lower than pH_PZC_, the surface is positively charged (Eq. (6)), otherwise, it is negatively charged (Eq. (7)). And nitrite ions, as anions, could be attached to the surface of catalysts which were positively charged. As a result, the low pH value could be beneficial for the adsorption of nitrite ions, thus improving the catalytic oxidation reaction rate.

6 $${\rm{MeOH}}+{H}^{+}\iff MeO{H}_{2}^{+}\,(pH < p{H}_{PZC})$$

7 $${\rm{MeOH}}+O{H}^{-}\iff Me{O}^{-}+{H}_{2}O\,(pH > p{H}_{PZC})$$

**Figure 3:**
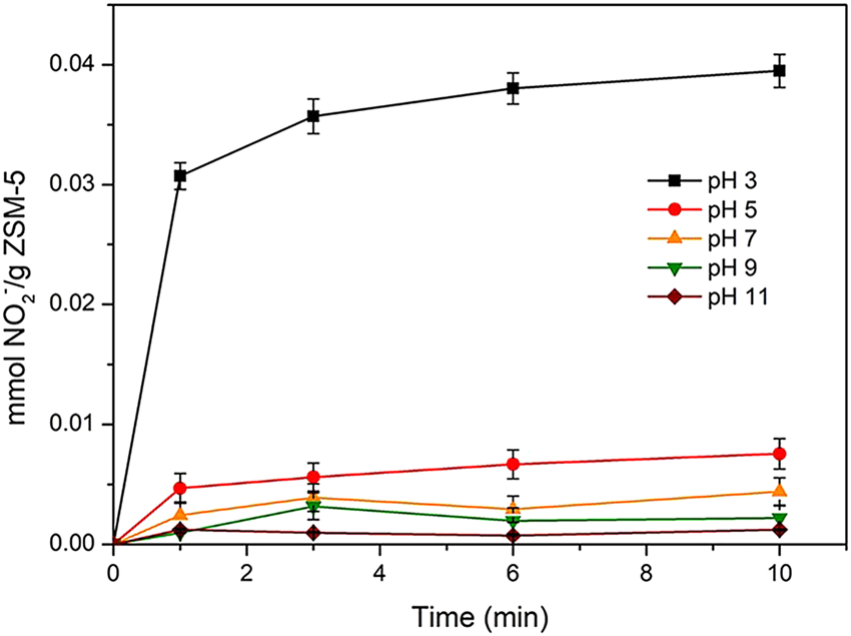
Adsorbed amounts of NO_2_^−^ on HZSM-5 zeolites at different pH values. Reaction conditions: C_0_ = 100 mg/L, T = 30 °C, catalyst dosage = 1.0 g, V = 200 ml, SiO_2_/Al_2_O_3_ = 360.

The results presented in Fig. 3 had confirmed that the adsorption capacity of nitrite ions on HZSM-5 zeolites at pH 3 was significantly higher than that at other pH values investigated. For instance, around 0.04 mmol of NO_2_^−^ was adsorbed on 1 g HZSM-5 zeolite within 10 min at pH 3, while less than 0.01 mmol/g adsorbed amounts was obtained at other pH values. The physical properties and points of zero charge for various HZSM-5 samples were given in Table 1. The pH_PZC_ of the HZSM-5 (SiO_2_/Al_2_O_3_ = 360) catalyst used in this test was found at around 5.20. Thus, the surfaces of ZSM-5 zeolites were positively charged at pH 3 and could adsorb large amounts of NO_2_^−^, resulting in the high oxidation efficiency. Furthermore, the pH value of the solution would also affect the stability of water-dissolved ozone. According to the literature^48^, the decomposition of ozone proceeds via various chain reaction steps, where the reaction rate constant under neutral condition was much lower than that under alkaline condition. The results shown in Supplementary Table S1 also confirmed that the concentration of aqueous ozone at pH 3 was found greater than that at higher pH values. This finding was consistent with the previous study^49^ suggesting ozone were more stable in acidic solution, which could promote the reaction involved with molecular ozone. Therefore, the oxidation of nitrite ions by ozone under acidic conditions might occur through the ozone molecular reaction pathway. Moreover, the slightly increased oxidation efficiency at pH 11 may be due to the hydroxyl radicals generated in the solution^50^.

**Table 1:** Surface area, pore structures and points of zero charge for HZSM-5 with different SiO_2_/Al_2_O_3_ ratios.

Sample (SiO_2_/Al_2_O_3_)	Surface area (m^2^ g^−1^)	Pore size (nm)	Pore volume (cm^3^ g^−1^)	pH_pzc_
18	171.5	2.99	0.13	4.04 ± 0.2
60	326.1	2.19	0.18	4.23 ± 0.3
130	390.6	2.27	0.22	5.37 ± 0.2
200	401.7	2.16	0.22	5.78 ± 0.2
360	412.6	2.22	0.22	5.20 ± 0.3

### Effects of SiO_2_/Al_2_O_3_ ratios

**Figure 4:**
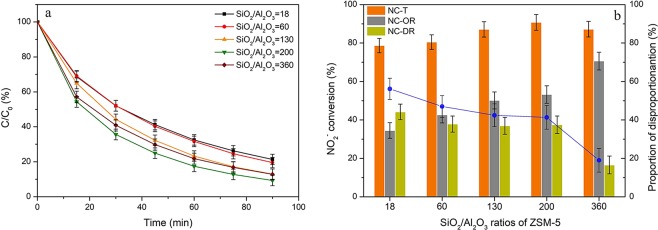
Effects of SiO_2_/Al_2_O_3_ ratios of HZSM-5 on (**a**) NO_2_^−^ content variations and (**b**) the related conversions via oxidation and disproportionation reactions. Reaction conditions: C_0_ = 100 mg/L, gas flow rate = 1.5 L/min, [O_3_] = 100 ppm, T = 30 °C, pH = 3, catalyst dosage = 0.2 g, V = 200 ml.

As shown in Fig. 4, NO_2_^−^ conversions on HZSM-5 zeolites in the presence of air and ozone was related to SiO_2_/Al_2_O_3_ ratios. The converting efficiency through oxidation of NO_2_^−^ catalyzed by HZSM-5 with relatively low SiO_2_/Al_2_O_3_ ratios (18, 60, 130 and 200) was 35%, 43%, 50% and 53%, respectively, while it was enhanced to 71% at SiO_2_/Al_2_O_3_ ratio of 360. The proportion of NO_2_^−^ disproportionation reaction was accordingly reduced with the increased silica contents. As shown in Supplementary Fig. S1, zeolites with high SiO_2_/Al_2_O_3_ ratios were of high adsorption capacity towards NO_2_^−^. The zeolites with high SiO_2_/Al_2_O_3_ ratios possessed relatively higher pH_PZC_ value (see Table 1), which would generate more positive charges at a certain pH value. Furthermore, the surface areas were increased at an elevated SiO_2_/Al_2_O_3_ ratio (Table 1). Both of the two aspects ensured the rising of adsorption capacity towards nitrite ions on the samples with high SiO_2_/Al_2_O_3_ ratios. Additionally, the previous studies^51,52^ have also found that water-dissolved ozone preferred to attach on the surface of HZSM-5 zeolites with high SiO_2_/Al_2_O_3_ ratios owing to less water adsorption. The results in Supplementary Fig. S2 further confirmed it that the adsorption of ozone on HZSM-5 zeolites was enhanced with higher SiO_2_/Al_2_O_3_ ratios. Therefore, both enhanced adsorption of nitrite ions and ozone would lead to the improved oxidation efficiencies of NO_2_^−^ for zeolites at higher silica content.

### Effects of HZSM-5 dosage and ozone concentration

**Figure 5:**
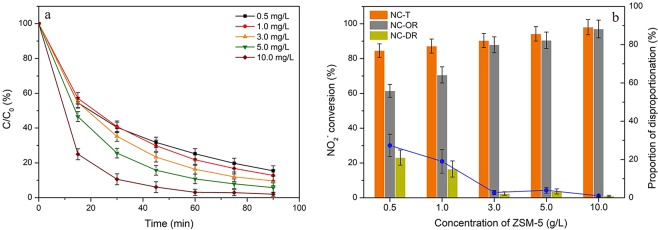
Effects of HZSM-5 dosage amount on (**a**) NO_2_^−^ content variations and (**b**) the related conversions via oxidation and disproportionation reactions. Reaction conditions: C_0_ = 100 mg/L, gas flow rate = 1.5 L/min, [O_3_] = 100 ppm, T = 30 °C, pH = 3, V = 200 ml, SiO_2_/Al_2_O_3_ = 360.

**Figure 6:**
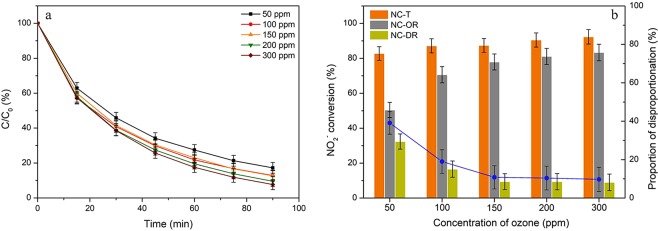
Effects of ozone concentration on (**a**) NO_2_^−^ content variations and (**b**) the related conversions via oxidation and disproportionation reactions. Reaction conditions: C_0_ = 100 mg/L, gas flow rate = 1.5 L/min, T = 30 °C, pH = 3, catalyst dosage = 0.2 g, V = 200 ml, SiO_2_/Al_2_O_3_ = 360.

The effects of HZSM-5 dosage and ozone concentration on the conversion of NO_2_^−^ through different reaction pathways were shown in Figs 5 and 6, respectively. It could be seen from Fig. 5 that the total conversion as well as the oxidation efficiency were enhanced with the increased HZSM-5 dosage. As expected, the rising of HZSM-5 dosage amount would facilitate the adsorption of ozone and nitrite ions, thereby increasing the oxidation efficiency over the surface of the catalysts. It was further confirmed that the surface reaction dominated the whole oxidation process compared to the reaction in the bulk solution. When the dosage amount increased over than 3 g/L, around 90% oxidation efficiencies could be observed and the disproportionation reaction of NO_2_^−^ barely occurred. These results revealed that the disproportionation reaction mainly happened in the bulk solution.

As presented in Fig. 6, the oxidation efficiency was improved from 50% to 71% as the ozone content increased from 50 ppm to 100 ppm, it maintained at about 80% as well as the proportion of disproportionation reaction remained at around 10% with further increased ozone concentration. Considering the concentration of ozone in solution was far from equilibrium^53^, it may suggest that the surface reaction was the main reaction pathway for nitrite oxidation by ozone. And the diffusion limitation of nitrite ions from aqueous phase to the surface of zeolites would somewhat inhibit further increase in the oxidation rate of NO_2_^−^ at an elevated ozone content since the adsorption of HZSM-5 towards ozone did not reach the saturation state^52^.

### Effects of oxygen partial pressure

**Figure 7:**
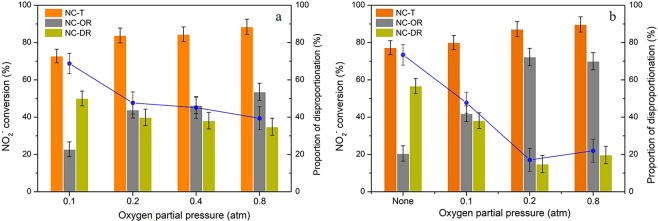
Effects of oxygen partial pressure on conversions of NO_2_^−^ (**a**) in absence of ozone and (**b**) in presence of ozone. Reaction conditions: C_0_ = 100 mg/L, gas flow rate = 1.5 L/min, [O_3_] = 100 ppm (if use), T = 30 °C, pH = 3, catalyst dosage = 0.2 g, V = 200 ml.

In order to clarify the role of oxygen and ozone in the oxidation of nitrite ions, the effects of oxygen partial pressure with/without ozone were then investigated. As shown in Fig. 7a, with oxygen alone, it could be seen that the oxidation efficiency of NO_2_^−^ was enhanced with an increased in oxygen partial pressure. However, when the oxygen partial pressure were higher than 0.2 atm, such enhancement was limited (the oxidation efficiency was less than 60%), which may attributed to the limited adsorbed amount of oxygen with the certain amount of zeolite dosage^43^. Moreover, considering quite low oxidation rate of NO_2_^−^ by oxygen in the bulk solution, the proportion of disproportionation reaction was still considerable even under high oxygen partial pressure. And from Fig. 7b, it could be also found that the conversion ratio via oxidization reaction still not very high (around 20%) with ozone alone. And in the presence of both oxygen and ozone, the oxidation efficiency was dramatically increased, which might suggest their synergistic effect. The existence of oxygen could improve the stability of ozone^54,55^ and ozone could hinder the disproportionation reaction in bulk solution.

### Proposed reaction mechanism

**Figure 8:**
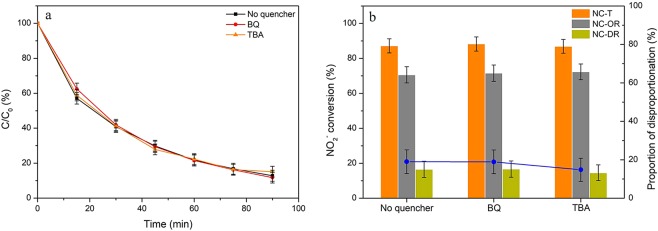
The variations in NO_2_^−^ concentration (**a**) and the related conversions via oxidation and disproportionation reactions (**b**) in the presence of BQ or TBA. Reaction conditions: C_0_ = 100 mg/L, gas flow rate = 1.5 L/min, [O_3_] = 100 ppm, T = 30 °C, pH = 3, catalyst dosage = 0.1 g, V = 200 ml, SiO_2_/Al_2_O_3_ = 360.

**Figure 9:**
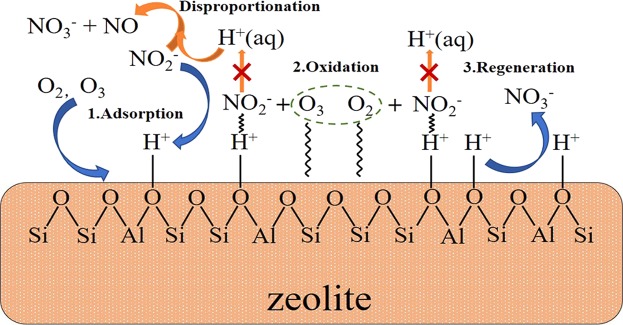
Proposed mechanism of catalytic oxidation of NO_2_^−^ on ZSM-5 zeolites.

Until now, it was very clear that the disproportionation reaction of NO_2_^−^ mainly happened in the bulk solution, while the occurrence of oxidation reaction would greatly inhibit it as a competitive reaction. And it could be concluded that the surface oxidation reaction was dominated in the total oxidation of nitrite ions, which could be verified by the fact that in the presence of HZSM-5, the oxidation efficiency was greatly improved (from less than 30% to around 70%) and the oxidation efficiency increased with the rising of HZSM-5 dosage. According to the experimental results regarding the effects of silica content and pH values, it was confirmed that the adsorption of nitrite ions played a vital role in surface catalytic oxidation reaction. The results at different temperature further verified it that the oxidation efficiency would decrease at higher temperature owing to the less adsorption of nitrite ions (see Figs S3 and S4). Nitrite ions can hardly be oxidized by oxygen in the bulk solution. And the presence of ozone could significantly enhance the oxidation rate of NO_2_^−^ both in the bulk solution and particularly on the zeolite surface under acidic conditions. Normally, the ozone-involved catalytic oxidation reaction proceeded via a radical (like hydroxyl radical) and/or direct molecular reaction pathways^56^. Based on the previous study^57^, nitrite ions are ready to react with hydroxyl radical to form nitrate ions. However, under acidic conditions, ozone is rather stable and hardly decomposes into hydroxyl radicals based on the analysis in Section 3.2. And the literatures^40,58^ also argued that the formation of radicals such as hydroxyl radicals and superoxide ions could not be promoted by zeolites. To further elucidate the reaction mechanism, the tests with the addition of radical scavengers were performed (As shown in Fig. 8), where p-benzoquinone (BQ) and tertiary butyl alcohol (TBA) were used as the scavengers to quench superoxide radicals and hydroxyl radicals, respectively^59^. It could find that the additions of BQ and TBA did not show evident effects on the oxidation reaction of nitrite ions. As such, the surface oxidation reaction on zeolite occurred through the molecular ozone reaction under pH 3. The proposed mechanism of the ozone-assisted catalytic oxidation of NO_2_^−^ was then presented in Fig. 9. Firstly, aqueous nitrite ions were adsorbed on the surface of HZSM-5 zeolites as well as oxygen and ozone. Then, the oxidation reactions proceeded among the adsorbed NO_2_^−^ and molecular oxidants.

## Conclusions

In this work, the reaction behaviors for ozone-assisted oxidation of NO_2_^−^ over various HZSM-5 zeolites had been investigated. It was found that the oxidation and disproportionation reactions of nitrite ions could take place simultaneously. And the disproportionation of NO_2_^−^ mainly happened in the bulk solution, while the occurrence of surface oxidation reaction would greatly inhibit it as a competitive reaction. The adsorption of nitrite ions played a vital role in surface catalytic oxidation reaction. In addition, high silica content and low pH value (especially at pH 3) would facilitate the surface oxidation reaction mainly owing to the enhanced adsorption of nitrite ions. The results in the presence of radical scavengers had not showed obvious changes, suggesting the catalytic oxidation of NO_2_^−^ on the zeolites could proceed through the direct reaction of molecular oxygen and ozone with adsorbed nitrite ions.

## Supplementary information

Supporting Information
