# Supplementary material for: Ozone-assisted catalytic oxidation of aqueous nitrite ions on HZSM-5 zeolites
Source: Sci Rep. 2019 Oct 4;9:14322. doi: 10.1038/s41598-019-50662-7 (PMC6778124; doi:10.1038/s41598-019-50662-7)
Supplement: Supplementary file 1 — Supporting Information [file 41598_2019_50662_MOESM1_ESM.pdf]

Ozone-assisted catalytic oxidation of aqueous nitrite ions on HZSM-5 zeolites

Mengyue Ying[1,2], Mengdi Zhang[1,2], Yue Liu[1,2]* and Zhongbiao Wu[1,2]

[1]Department of Environmental Engineering, Zhejiang University, Hangzhou 310027, P. R. China

[2]Zhejiang Provincial Engineering Research Center of Industrial Boiler & Furnace Flue Gas Pollution Control, Hangzhou 310027, P.R. China

*Corresponding author: Tel: +86 571 87953088; Fax: +86 87953088.

E-mail address: yueliu@zju.edu.cn

Stability experiments

The stability of water-dissolved ozone at different pH was investigated via following processes. The ozone flow (0.6 mg/min) was bubbled into the solution with different pH value (3, 5, 7, 9 and 11) for 15 min at the temperature of 30 °C and then the samples were collected to determine the concentration of aqueous ozone by measuring at 253.7 nm via the UV-VIS spectrophotometer (UV-2600, SHIMADZU).

Adsorption experiments

For NO_2^- adsorption experiments, 1 g zeolites with different SiO_2/Al_2O_3 ratios (18, 60, 130, 200 and 360) were added into 200 ml solution (100 mg/L NO_2^-) with pH value of 3, respectively. The mixture was continuously stirred for 1 min and then was rested for another 9 min at 30 °C. Samples were collected at 1, 3, 6 and 10 min followed by filtration (PTEE 0.22 μL springe filter).

For aqueous ozone adsorption experiments, the pH of ultra-pure water was adjusted to 3 in order to improve the stability of dissolved ozone in water. The ozone flow (0.60 mg/min) was bubbled into the solution (200 ml) for 15 min at 30 °C, and then the solution was sampled (40 ml of the solution was drawn). Subsequently, 2 g ZSM-5 zeolites with different SiO_2/Al_2O_3 ratios (18, 60, 130, 200 and 360) were added into the remaining solution (160 ml), respectively. The mixture was then stirred for 30 s and was rested for another 30 s, finally the samples were collected and then were filtered (PTEE 0.22 μL springe filter) before analysis

Effects of temperature

Fig. S3 showed the effect of temperature on NO_2^- conversions through different ways.

The total conversion of NO_2^- was enhanced with the increase in temperature. However, the oxidation efficiency was decreased and the proportion of disproportionation was increased with temperature from 20 to 50 °C, which could be attributed to the inhibition of NO_2^- adsorption on the catalyst surface (see Fig. S4) at higher temperature. The less adsorption of NO_2^- would results in higher amount of NO_2^- ions in the bulk solution. And the interaction between free nitrite ions and hydrogen ions was accelerated by the increase of temperature, thus leading to the enhanced proportion of disproportionation reaction. The results further suggested that the oxidation reaction mainly occurred on the catalyst surface and the adsorption of nitrite ions played a vital role in surface catalytic oxidation reaction.

Table S1 The stability of water-dissolved ozone at different pH values

pH values	3.0	5.0	7.0	9.0	11.0
Concentration of aqueous O_3 (mg/L)	1.967 ± 0.016	1.631 ± 0.032	1.151 ± 0.016	1.151 ± 0.032	0.016 ± 0.016

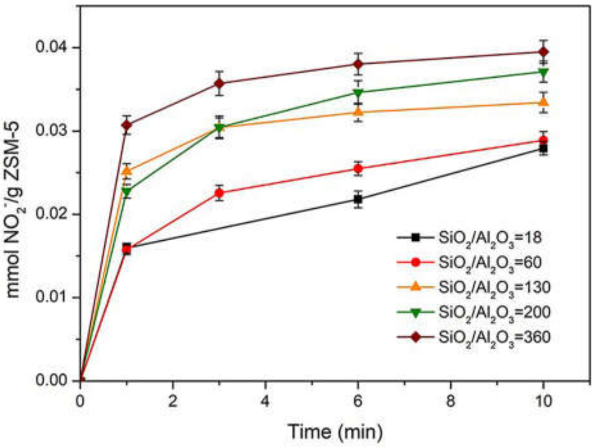

Fig. S1 Adsorbed amounts of NO_2^- on HZSM-5 zeolites with different SiO_2/Al_2O_3 ratios.

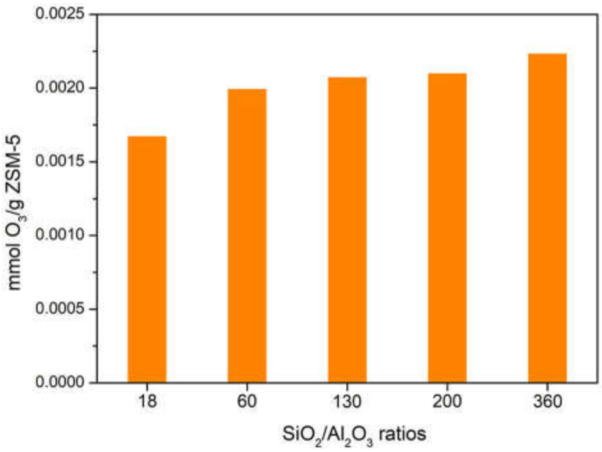

Fig. S2 Adsorbed amounts of ozone on HZSM-5 zeolites with different SiO_2/Al_2O_3 ratios.

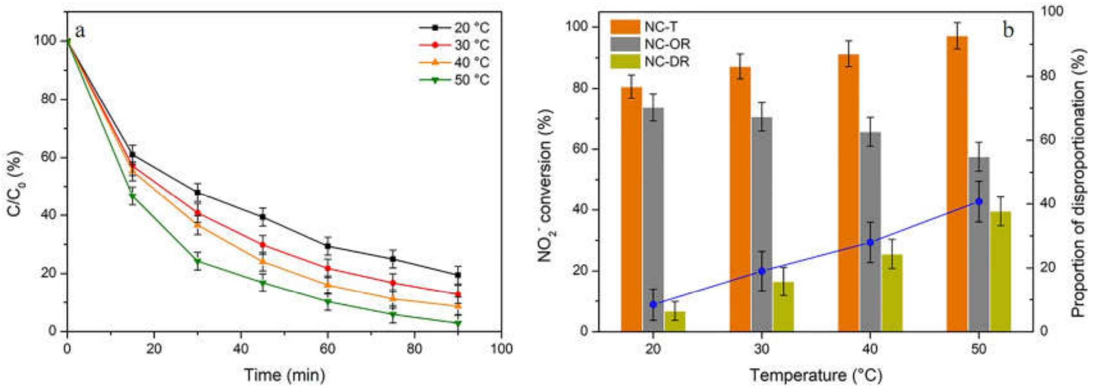

Fig. S3 Effects of temperature on (a) NO_2^- content variations and (b) the related conversions via oxidation and disproportionation reactions. Reaction conditions: C_0 = 100 mg/L, gas flow rate = 1.5 L/min, $[O_3]$ = 100 ppm, pH=3, catalyst dose = 0.2 g, V = 200 ml, SiO_2/Al_2O_3 = 360.

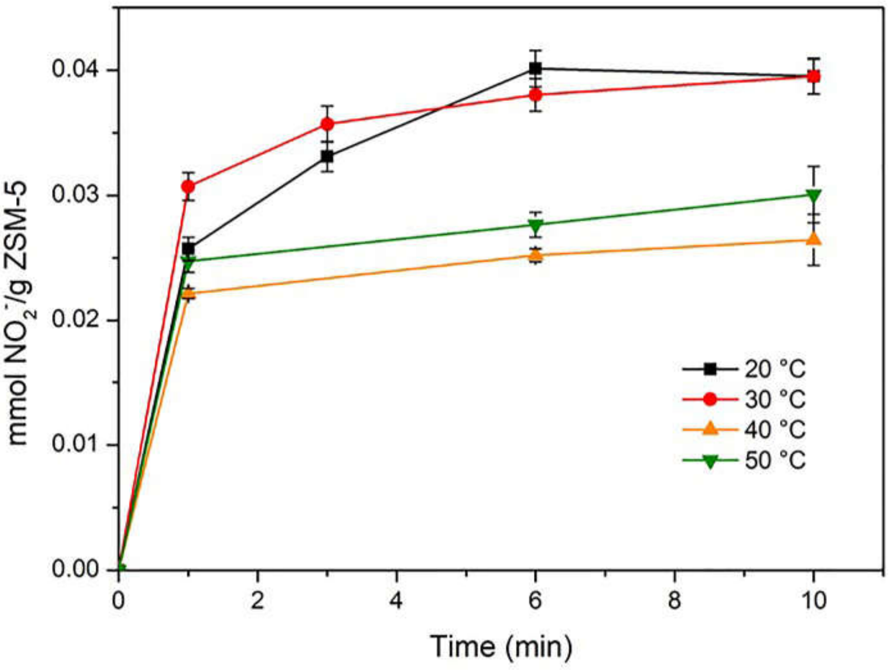

Fig. S4 Adsorbed amounts of NO_2^- on HZSM-5 zeolites under different temperature.

Reaction conditions: C_0 = 100 mg/L, pH=3, catalyst dose = 1.0 g, V = 200 ml,

SiO_2/Al_2O_3 = 360.
